# Neuropsychological dysfunction associated with cancer and cancer therapies: a conceptual review of an emerging target

**DOI:** 10.1038/sj.bjc.6601772

**Published:** 2004-04-06

**Authors:** J S Wefel, A E Kayl, C A Meyers

**Affiliations:** 1 Department of Neuro-Oncology, The University of Texas MD Anderson Cancer Center, 1515 Holcombe Blvd., Unit 431, Houston, TX 77030-4009, USA; 2 The Brain Tumor Center, The University of Texas MD Anderson Cancer Center, 1515 Holcombe Blvd., Unit 431, Houston, TX 77030-4009, USA

## Abstract

Neuropsychological dysfunction associated with cancer and cancer treatment is a growing concern. Methodological limitations permeate the corpus of research in this area and have limited our understanding of the multifactorial nature of this process. The following review provides a summary of the current state of knowledge and highlights future directions.

While it is generally recognised that central nervous system (CNS) cancer and many of the therapeutic modalities used to treat cancer can cause alterations in neurocognitive function, our knowledge about the nature, severity, and course of neurocognitive dysfunction is limited. Traditionally, treatment outcome has focused on the length of survival and neurological or physiological changes such as peripheral neuropathy, ototoxicity, or encephalopathy rather than indices of ‘quality of life’, such as neurocognitive function. Neurocognitive function has been demonstrated to be a sensitive, viable, and important end point that measures clinical benefit on patient functioning that is not adequately captured in clinical trials with measures of overall survival or patient performance status (Meyers and Hess, 2003). In the United States, government agencies have emphasised the need to develop and utilise multifaceted end points in clinical trials that will measure disease-related symptoms and/or quality of life. In addition to monitoring neurotoxicity, neurocognitive function has been demonstrated to be a sensitive predictor of patient survival (Meyers *et al*, 2000b), and change in neurocognitive function has been found to precede MRI evidence of tumour recurrence in glioma patients (Armstrong *et al*, 2003; Meyers and Hess, 2003). Such findings have prompted the incorporation of neuropsychological evaluations in the clinical care of cancer patients (Meyers, 1997).

Advances in the successful treatment of cancer have been achieved largely by an increased aggressiveness of therapy, which now generally combines surgery, radiation, cytotoxic drugs, and immunotherapy. Unfortunately, cancer treatments are not highly specific and place normal tissues and organs at risk. The CNS is vulnerable to many types of cancer treatments, both systemic and those directed against CNS tumours. In addition, many adjuvant medications necessary for the treatment of medical complications also affect CNS function (e.g. steroids, antiepileptics, immunosuppressive agents, and drugs used for pain, nausea, and infection).

There is a burgeoning literature on the neurocognitive effects of cancer treatment. Unfortunately, few methodologically rigorous studies exist to guide clinical practice. Most studies are retrospective, fail to incorporate assessments of pretreatment neurocognitive and neurobehavioural function, consist of small and heterogeneous samples, lack appropriate control groups, and suffer from poor measurement selection. Owing to the state of the literature, we will first describe principles that we believe underlie specific areas of practice and research. The empirical findings that support these principles will then be reviewed to provide a summary of the current knowledge with regard to neurocognitive dysfunction. These principles provide clinicians and researchers with a starting point from which further refinement of these concepts are expected. Finally, we will highlight key issues that may improve future research and patient care.

## RADIOTHERAPY

Principle: *Radiotherapy, whether incidental or directed principally at brain tissue, produces a predictable pattern of neurocognitive and neurobehavioural alterations. The development of these features and the time course are strongly related to treatment parameters, concomitant adjuvant therapy, and patient characteristics.*

The adverse effects of radiation to the brain, both as primary CNS therapy and prophylactic treatment, have been previously reviewed in detail (Crossen *et al*, 1994; Keime-Guibert *et al*, 1998). The development of neurologic and/or neuropsychological dysfunction is often the greatest dose-limiting factor of radiotherapy (XRT). Pathologically, autopsy reports have suggested that radiotherapy primarily affects the white matter tracts and cerebral vasculature of the brain via two mechanisms: (1) damaging oligodendrocytes, thereby creating axonal demyelination and (2) disrupting vascular endothelial cells contributing to coagulative necrosis, vessel wall thickening, and focal mineralisation. Owing to the relative density of white matter in frontal and subcortical areas, cognitive impairments consistent with frontal network systems dysfunction are common, including impaired processing speed, attention (e.g. working memory), learning efficiency and memory retrieval, executive function (e.g. mental flexibility), and often bilateral decline in motor function (e.g. fine motor dexterity) (Crossen *et al*, 1994; Gregor *et al*, 1996; Meyers *et al*, 2000a).

The occurrence of radiation encephalopathy has been most well studied in patients receiving either conventional, hyperfractionated, or whole brain radiotherapy. The effects of stereotactic radiosurgery and intensity modulated radiotherapy are currently unknown. Radiation encephalopathy has been separated into three stages: acute reaction, early-delayed reaction, and late-delayed reaction (Sheline, 1977). Radiation to the brain is rarely administered without systemic chemotherapy for the primary disease, and it is often not possible to separate the adverse effects of radiation from chemotherapy. The toxicity of radiation is likely synergistic with concurrent chemotherapy (Crossen *et al*, 1994). Thus, discussion of treatment effects will assume that the toxicity is caused primarily by cranial irradiation, although the possible synergistic toxicity of multimodality therapy is yet to be fully delineated. Risk factors for developing XRT-induced cognitive dysfunction and radiation necrosis include age >60 years old, >2 Gy dose per fraction, higher total dose, greater volume of brain irradiated, hyperfractionated schedules, shorter overall treatment time, concomitant or subsequent use of chemotherapy, and presence of comorbid vascular risk factors (e.g. diabetes) (Crossen *et al*, 1994; Lee *et al*, 2002).

A transient acute encephalopathy, resulting in generalised neurocognitive dysfunction, has been described, which is thought to be related to breakdown of the blood–brain barrier and is occasionally associated with focal neurologic signs, suggesting recurrent neoplasm (Crossen *et al*, 1994). However, the incidence of early-delayed effects of radiotherapy has been reduced with corticosteroid therapy. Studies of the neurocognitive functioning of patients surviving more than a year postradiotherapy have yielded conflicting results. Meyers *et al* (2000a) reported on a cohort of patients who received paranasal sinus radiation between 20 months and 20 years prior. Neuropsychological test results revealed 80% of the patients exhibited impaired memory, approximately 33% manifested slowed visuomotor speed, executive dysfunction, and poor fine motor dexterity. Others have failed to find significant late-delayed neurocognitive dysfunction as a result of radiotherapy (Vigliani *et al*, 1996; Torres *et al*, 2003). Differences in reported radiotherapy-associated cognitive dysfunction (incidence estimates that vary from 0 to 86%) may in part be related to differences in treatment variables, study methodology, and the disease that is being treated.

## CHEMOTHERAPY

Principle: *Adjuvant chemotherapy has been associated with decrements of neurocognitive and neurobehavioural functioning during the acute phase, but the persistence of these sequelae remain controversial.*

Although chemotherapy has proven beneficial in the treatment of a variety of malignancies, these treatments may have both acute and persistent adverse effects on the nervous system (Keime-Guibert *et al*, 1998). A variety of nonspecific neurological complications associated with chemotherapy have been described, including: (1) an acute encephalopathy characterised by a confusional state, insomnia, and often agitation, which is commonly believed to resolve off treatment; (2) chronic encephalopathy characterised by cognitive dysfunction consistent with a ‘subcortical dementia’, incontinence, and gait disturbance; (3) stroke-like episodes associated with transient motor impairments; (4) a cerebellar syndrome with symptoms ranging from ataxia to a pancerebellar syndrome; and (5) a variety of peripheral neuropathies.

Certain agents are known to be particularly neurotoxic. For instance, methotrexate and 5-FU can cause diffuse white matter changes on neuroimaging. Other agents have been found to affect specific neuroanatomical structures preferentially. For example, CI-980 selectively affects memory by binding to tubulin at the colchicine binding site and selectively blocking choline acetyltransferase in the hippocampus and basal forebrain (Meyers *et al*, 1997).

Reports of neurophysiologic and functional neuroimaging abnormalities in breast cancer survivors previously treated with adjuvant chemotherapy have also been observed. However, the relationship between these indices of brain function and neurocognitive function is not one-to-one. Schagen *et al* (2001) examined event-related potentials, quantitative electroencephalography, and neurocognitive function approximately 2 years after chemotherapy in women with breast cancer who received high, standard, or no chemotherapy. They found asymmetry of the alpha rhythm in a subset of the patients who previously received chemotherapy that was not associated with neurocognitive test results or emotional distress. Silverman *et al* (2003) examined the relationship between regional cerebral metabolism in breast cancer survivors. Women who had previously received chemotherapy alone evidenced hypometabolism in the superior frontal gyrus of the dorsolateral prefrontal cortex as well as Broca's area and its homologous counterpart in the nondominant hemisphere. Further, women who received tamoxifen (TAM) in addition to chemotherapy evidenced even greater hypometabolism.

A recent meta-analysis examining the neurocognitive sequelae of chemotherapy in adults reported that compared to normative data, control samples, or baseline test performance, patients receiving adjuvant chemotherapy experienced declines in six out of seven neurocognitive domains evaluated (i.e. attention, processing speed, verbal memory, visuospatial, executive and motor function). Memory and executive function reached statistical significance and demonstrated a rather large effect size (Cohen's *d* approximately 0.9). Motor function exhibited a smaller effect size (approximately 0.5), but also reached statistical significance. Importantly, when only studies that used longitudinal designs incorporating baseline evaluations were examined, none of the cognitive domains reached significance and all demonstrated only modest effect sizes (Anderson-Hanley *et al*, 2003).

Longitudinal investigations that measure patient's baseline neurocognitive and neurobehavioural function prior to adjuvant therapy are required to measure idiographic change in function and to parse out neurocognitive impairment caused by the disease from that caused by the treatment. For example, Meyers *et al* (1995a) demonstrated that a 70–80% of patients with small-cell lung cancer have memory deficits, 38% have deficits in executive functions, and 33% have impaired motor coordination *before* treatment is initiated.

Several centres (McAllister *et al*, 2000; Fliessbach *et al*, 2003) have demonstrated the ability to deliver potentially neurotoxic therapies without inducing neurocognitive dysfunction. These prospectively designed trials of treatment protocols involving intravenous, intra-arterial, or intraventricular multiagent chemotherapy with blood–brain barrier disruption for the treatment of primary CNS lymphoma reported no significant neurocognitive dysfunction in patients who achieve a durable remission 1 year after treatment. Despite limitations in our understanding of chemotherapy-related neurotoxicity, there has been growing concern that subgroups of patients develop iatrogenically produced neurocognitive dysfunction that can be disabling in severity.

## BIOIMMUNOTHERAPY

Priniciple: *Biologic response modifiers are frequently associated with both acute neurobehavioural and neurocognitive alterations. Exogenous treatment with proinflammatory cytokines contributes to alterations of neurotransmitter systems, hypothalamic-pituitary–adrenal axis endocrine function, and secondary messengers. However, the persistence of these untoward effects and the efficacy of treatments that limit or prevent these effects are poorly understood.*

Proinflammatory cytokines have been reported to have both direct and indirect effects on CNS function through the alteration of neurotransmitters, neuroendocrine function, and induction of secondary cytokine activity. These cascades produce a host of neurobehavioural sequelae that have been termed ‘sickness behaviour’ (Kelley *et al*, 2003). Symptoms of sickness behaviour include fever, weakness, malaise, listlessness, and concentration difficulties. In addition, more than 50% of patients receiving cytokine therapy have documented neurocognitive impairments (Meyers and Abbruzzese, 1992). For a review of these agents, their mechanisms of action, and potential treatment strategies refer to Meyers and Valentine (1995b) and Trask *et al* (2000).

Patients who manifest neurotoxicity subsequent to endogenous administration of cytokines develop neurocognitive deficits that are consistent with frontal network systems dysfunction including diminished information processing speed and simple reaction time, attentional and executive dysfunction, reduced learning and memory, impaired fine motor dexterity, and neurobehavioural sequelae including ‘sickness behaviour’, depression, and anxiety (Valentine *et al*, 1998; Trask *et al*, 2000). Investigations utilising functional neuroimaging have demonstrated abnormalities in frontal regions that parallel the neurocognitive findings (Meyers *et al*, 1994; Juengling *et al*, 2000).

The neurovegetative and somatic symptoms associated with IFN-*α* neurotoxicity have been reported to occur within the first 2 weeks of treatment, whereas the cognitive and mood symptoms often develop within 8–12 weeks after initiating treatment (Capuron *et al*, 2001). This observation is consistent with other reports that the length of treatment, dose, and route of administration appear to be key factors related to the development of neurotoxicity (Meyers, 1997). Although these symptoms may persist for a small subgroup, most can be successfully palliated with prophylactic or symptomatic antidepressant therapy for neurobehavioural symptoms (Musselman *et al*, 2001), stimulant therapy for fatigue and neurobehavioural slowing, and opiate antagonist therapy for cognitive disorders (Valentine *et al*, 1998).

## HORMONAL THERAPY

Principle: *Abrupt alteration of an individual's hormonal milieu has been associated with neurocognitive and neurobehavioural impairments. The effects of more insidious and less direct hormonal alterations are largely unknown.*

Oestrogen receptors have been discovered in many areas of the brain important for cognitive functioning including the hypothalamus, anterior pituitary, amygdala, and CA1 of the hippocampus (McEwen and Alves, 1999). Human and animal studies (Yaffe *et al*, 1998) have elucidated several possible mechanisms through which oestrogen affects neurocognitive and neurobehavioural function including: (1) increasing cholinergic activity through its actions on choline acetyltransferase; (2) maintenance of dendritic spine density on CA1 pyramidal cells of the hippocampus; (3) facilitating induction of long-term potentiation in the hippocampus; (4) increasing serotonergic and cholinergic activity, thereby maintaining neural circuitry; (5) altering lipoprotein; and (6) decreasing the risk of cerebral ischaemia.

The effects of hormonal challenges in women (e.g. antioestrogens) have been examined with both neuroimaging (Berman *et al*, 1997) and neurocognitive probes (Varney *et al*, 1993, Rich and Maki, 1999). In summary, a pattern of relative hypometabolism in prefrontal cortex has been demonstrated with PET, and neurocognitive impairments in memory, executive function, and motor coordination have been reported. The severity of these impairments varies, but has occasionally been reported to result in impairments of daily living and vocational function.

Tamoxifen is a widely used selective oestrogen receptor modulator (SERM) for the treatment of breast cancer. It is estimated that approximately 11% of women will develop breast cancer in their lifetime. Moreover, in the United States, it is estimated that based on year 2000 census data more than 2 million women could benefit from prophylactic use of TAM (Freedman *et al*, 2003), highlighting the importance of understanding the potential neurocognitive side effects of this agent. TAM is known to have both agonist and antagonist effects in the periphery and in the brain (McKenna *et al*, 1992). It has also been reported to influence the production of proinflammatory cytokines (IL-1, IL-6, and TNF) that are associated with cognitive dysfunction (Järvinen *et al*, 1996). Retrospective investigations examining the neurotoxic effects of chemotherapy on neurocognitive function in breast cancer patients have not found differences between women who either received or did not receive TAM subsequent to chemotherapy (Schagen *et al*, 1999). However, PET imaging has demonstrated greater prefrontal hypometabolism in women with treatment histories that included both chemotherapy and TAM compared to women treated with chemotherapy alone (Silverman *et al*, 2003).

Paganini-Hill and Clark (2000) reported that women who previously used TAM performed similarly on a neurocognitive screen when compared to a group of breast cancer survivors never exposed to TAM, while current TAM users had slightly less complex narrative writing samples. However, this methodology is ineffective for examining cognitive function and potentially yields useless data. We have been engaged in a prospective, longitudinal trial utilising a comprehensive neuropsychological assessment to determine the neurocognitive and neurobehavioural sequelae associated with adjuvant TAM therapy. Our unpublished preliminary findings suggest that a subgroup of women taking TAM experience a significant neurotoxicity consisting of memory, executive, and motor dysfunction associated with increased affective distress, decreased QOL, and diminished ability to maintain productive activities. This trial is also examining potential mechanisms responsible for this neurotoxicity including alterations in circulating levels of proinflammatory cytokines as well as fluctuations in stress and sex hormones. A number of other SERMs and aromotase inhibitors are currently being investigated for clinical use and may also be associated with similar reports of neurotoxicity.

Testosterone supplementation has been reported to enhance cognitive function in healthy older men (Cherrier *et al*, 2001). The hippocampus contains testosterone receptors as well as estradiol receptors, and thus it is unclear if these beneficial effects arise through the androgen receptors or via aromitisation to estradiol, or both. Hormonal challenges in men via administration of luteinising hormone-releasing hormone agonists, such as leuprolide or goserelin, also may adversely affect hippocampal function through these hormonal channels. There are inconsistent findings with regard to the safety profile of androgen ablating agents, with some authors reporting no evidence of neurocognitive decline or neurobehavioural dysfunction (Salminen *et al*, 2003), and others finding impaired memory, attention, and executive function (Green *et al*, 2002).

## ADJUVANT MEDICATIONS AND MEDICAL COMPLICATIONS

Priniciple: *The assessment of cognitive dysfunction secondary to cancer treatment is complicated by the use of supportive medications (e.g. steroids, immunosuppressive agents, anticonvulsants) that can alter cognitive function.*

In addition to the neurotoxic effects of primary cancer therapy, adjuvant medications such as steroids, anticonvulsants, and pain medications may also cause neurocognitive and neurobehavioural symptoms. The use of glucocorticoids is ubiquitous and is associated with a 5–50% incidence of steroid-induced psychiatric syndromes including euphoria, mania, insomnia, restlessness, and increased motor activity. Glucocorticoids have been implicated in the development of memory dysfunction across a variety of conditions including chronic stress and post-traumatic stress disorder. Certain anticonvulsants (e.g. topiramate, phenobarbital) are also known to have adverse neurocognitive effects. Both seizure frequency and the use of anticonvulsants have been demonstrated to adversely impact neurocognitive function in brain tumour patients (Klein *et al*, 2003). Pharmacologic intervention for symptoms of pain may cause sedation and associated diminution of neurocognitive function.

Abnormalities in endocrinologic function secondary to hypothalamic/pituitary injury are very common following radiotherapy. Thyroid dysfunction, loss of libido, and erectile dysfunction are present in a large proportion of patients. Endocrinologic replacement therapy has the potential to improve neurocognitive and neurobehavioural function in patients who have abnormal hormone levels. Anaemia is a side effect of some chemotherapeutic regimens that is associated with both fatigue and neurocognitive dysfunction. Epoetin alpha therapy has been found to minimise neurocognitive decline in breast cancer patients receiving anthracycline-based chemotherapies relative to placebo (O'Shaughnessy, 2002). The aetiology of cancer-related fatigue is likely multifactorial and includes anaemia, cachexia, systemic illness, pain, and medications (Kurzrock, 2001). Both peripheral and central factors associated with cytokine production may be involved in the development and maintenance of this state.

## NEUROPSYCHOLOGICAL ASSESSMENT

**Figure 1:**
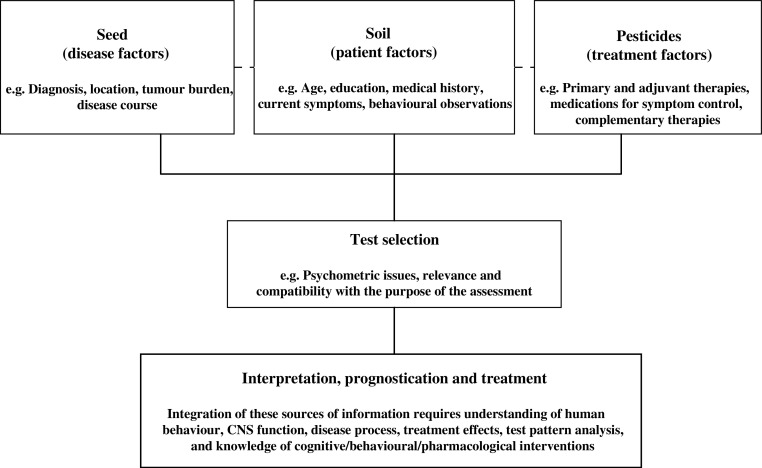
Considerations for the cognitive assessment of the oncology patient.

Although the importance of cognitive evaluations in the care of cancer patients and in clinical cancer trials is receiving greater recognition, assessment methods remain less than optimal in most cases. Cognitive assessment is a complex undertaking that requires diverse skills. Although the administration of tests is a relatively simple endeavour, interpretation of test scores relies heavily on the clinician's interviewing skills, appreciation of social and cultural factors, understanding of test construction and psychometrics, psychodiagnostic skills, and knowledge of the human nervous system (see Figure 1

).

The contemporary scientific literature is cluttered with poorly designed studies that may lead investigators and the readership to incorrect conclusions. Clinicians and researchers must keep a few basic principles in mind when developing a plan for assessment. First, test selection will vary depending on the question under consideration. Second, the measures chosen should have alternate forms or be relatively resistant to practice effects, characteristics that are especially important if one plans to test patients repeatedly. Third, selected measures should be psychometrically sound, with established reliability and validity, and appropriate normative studies. Finally, it is important to select measures that are sensitive to subtle changes in cognitive function often experienced by patients with cancer. Attention, processing speed, learning/memory functions, and motor skills are particularly vulnerable and should be carefully evaluated for signs of dysfunction.

## SUMMARY

Cancer is becoming a chronic illness, requiring on-going symptom assessment and intervention. The number of long-term cancer survivors will continue to increase as will the number of survivors with neurocognitive and/or neurobehavioural impairment. It is important to note that treatment-related cognitive decline is not universal among cancer patients. Some individuals are able to tolerate treatment with little physical discomfort and no obvious neurocognitive impairments, while others will develop significant toxicities that seriously compromise their perceived quality of life and prevent them from resuming their usual social and occupational roles. However, any adverse effects of cancer treatment must always be considered in the light of potential therapeutic benefits.

The nature of neurocognitive and neurobehavioural dysfunction is yet to be fully characterised. Methodological challenges have plagued research in this area and seemingly contradictory findings saturate the existing literature. Increased inclusion of comprehensive neuropsychological evaluations in clinical research will further our understanding of the nature, severity, and processes underlying neurocognitive dysfunction in the patient with cancer. Multidisciplinary investigations are essential. Utilising advances in neuropsychology, cognitive neuroscience, genomics, proteonomics, molecular epidemiology, functional neuroimaging, neuroimmunology, and traditional oncologic disciplines will ultimately contribute to understanding the relationships between disease, treatment, and patient factors in the manifestation of altered neurocognitive and neurobehavioural function. These multidisciplinary investigations will identify which agents are most neurotoxic in the context of different treatment regimens, the course of the neurocognitive and neurobehavioural dysfunction, the cognitive and neurobehavioural domains most affected, the mechanisms for these effects, the host risk factors that create a diathesis for the expression of this neurotoxicity, and which neuroprotective or rehabilitative therapies may be most efficacious in preventing or treating these adverse symptoms.
